# Association between household coal use duration and upper gastrointestinal cancer and precancerous lesions: a cross-sectional study

**DOI:** 10.3389/fpubh.2026.1878399

**Published:** 2026-06-30

**Authors:** Kai Xu, Zehua Wang, Xia Guo, Peng Cui, Lizhong Fan, Hengbo Jia, Wenqing Hu

**Affiliations:** 1 Changzhi People's Hospital Affiliated to Shanxi Medical University, Changzhi, Shanxi, China; 2 Department of Clinical Discipline Construction Centre, Shanxi Medical University, Taiyuan, Shanxi, China; 3 Institute for the Prevention and Treatment of Upper Gastrointestinal Tumors, Shanxi Medical University, Changzhi, Shanxi, China; 4 Heping Hospital Affiliated to Changzhi Medical College, Changzhi, Shanxi, China

**Keywords:** cross-sectional study, household coal use, indoor air pollution, precancerous lesions, upper gastrointestinal cancer

## Abstract

**Background:**

To explore the association between household coal use duration and upper gastrointestinal cancer and precancerous lesions.

**Methods:**

This study is a cross-sectional study. The research subjects were selected from those in Lucheng District and Pingshun County of Shanxi Province who participated in the upper gastrointestinal cancer screening and had complete data. The demographic characteristics, disease history, lifestyle, and household coal usage were collected through standardized questionnaires. A multivariable logistic regression model was used to assess the association between the duration of household coal usage and upper gastrointestinal cancer and precancerous lesions. Subgroup analysis was conducted based on age, gender, region, smoking status, and alcohol consumption. Sensitivity analysis was used to verify the robustness of the results.

**Results:**

A total of 55,820 participants were included, including 783 cases with upper gastrointestinal cancer and precancerous lesions and 55,037 controls. After adjustment for potential confounders, a positive association was observed between the duration of household coal use and upper gastrointestinal cancer and precancerous lesions (OR = 1.13, 95% CI: 1.05–1.24, *P* = 0.003). Stratified analyses showed statistically significant associations among women, individuals aged ≥65 years, non-smokers, non-drinkers, and residents of Pingshun County. Nevertheless, interaction tests were not statistically significant across all subgroup variables (all *P* for interaction > 0.05), indicating no evidence of effect modification. Sensitivity analyses demonstrated the stability and robustness of the observed association.

**Conclusions:**

A positive association was observed between the duration of household coal use and upper gastrointestinal cancer and precancerous lesions. Given the cross-sectional nature of this study, causal inferences cannot be established. Prospective studies are warranted to further validate these findings.

## 1 Introduction

Upper gastrointestinal cancer (mainly including esophageal cancer and gastric cancer) is a common malignant tumor worldwide and is characterized by high incidence and high mortality (1, 2). In 2022, there were 1.479 million new cases and 1.105 million deaths from upper gastrointestinal cancer globally, accounting for approximately 7.5% and 11.4% of all cancer incidence and mortality, respectively (3). As a high-incidence province for upper gastrointestinal cancer in China, the 2022 Shanxi Cancer Registry Annual Report showed (4, 5) that the age-standardized incidence and mortality rates of upper gastrointestinal cancer, mainly esophageal cancer and gastric cancer, in Shanxi Province were significantly higher than the national and global averages, indicating a severe disease prevention and control situation and an urgent need to identify locally modifiable risk factors. As an important coal-energy production base in China, rural and county residents in Shanxi Province have long relied extensively on coal as the primary fuel for household heating and cooking (6). Previous studies have mainly focused on the association between household coal use exposure and respiratory diseases or lung cancer (7); however, accumulating epidemiological evidence in recent years has suggested a significant association between coal-related indoor air pollution and the risk of digestive tract tumors (8). Multiple carcinogenic substances released during coal combustion, such as polycyclic aromatic hydrocarbons and fine particulate matter, may exert their effects not only through the respiratory system but also through dual pathways involving local swallowing exposure and systemic blood circulation, thereby continuously stimulating and damaging the upper gastrointestinal mucosa and increasing the risk of carcinogenesis and precancerous lesions in local tissues (9). Although previous studies have suggested an association between indoor coal exposure and the risk of upper gastrointestinal cancer, most studies have characterized exposure using crude indicators such as whether coal was used, which are insufficient to reflect long-term cumulative effects, and localized studies in coal-rich and high-incidence regions for upper gastrointestinal cancer, such as Shanxi Province, remain limited (6, 8, 10). Against this background, based on cross-sectional data from 55,820 participants undergoing upper gastrointestinal cancer screening in Changzhi, Shanxi Province, this study adopted a cross-sectional design to systematically investigate the association between duration of household coal use and upper gastrointestinal cancer and precancerous lesions, aiming to provide a scientific basis for localized prevention and control strategies for upper gastrointestinal cancer in high-incidence areas of Shanxi Province.

## 2 Subjects and methods

### 2.1 Study participants

This study was a population-based cross-sectional study, and the data were derived from the 2025 upper gastrointestinal cancer early screening, early diagnosis, and early treatment program of the “Healthy Changzhi Promotion Action” conducted in Changzhi, Shanxi Province. The source population of the study consisted of permanent residents of Pingshun County and Lucheng District who completed questionnaire surveys during the program period. According to the annual implementation plan of the program, Pingshun County and Lucheng District were the designated screening areas for 2025; therefore, this study included eligible permanent residents from these two regions who completed questionnaire surveys during the program period. In addition, both regions are high-incidence areas for upper gastrointestinal cancer in Shanxi Province, meeting the representativeness requirements for the disease population in this study. Meanwhile, the two regions differ significantly in household coal use rate, socioeconomic level, proportion of rural population, and coverage of upper gastrointestinal cancer early screening, thereby providing a reasonable basis for subgroup stratified analyses. Therefore, these two regions were ultimately selected as the study sites. The inclusion criteria were as follows: (1) age 50–75 years; (2) permanent residents of Pingshun County or Lucheng District, Changzhi City (residence duration ≥6 months); and (3) completion of a complete questionnaire survey. The exclusion criteria were as follows: (1) missing key information; and (2) obvious logical errors or abnormalities in the survey data that could not be verified.

Ethical approval was obtained from the Ethics Committee of Changzhi People's Hospital Affiliated to Shanxi Medical University (Approval No. 2026K007), and informed consent was obtained from all participants.

### 2.2 Case definition

#### 2.2.1 Case group

The study outcome was defined as a composite endpoint of upper gastrointestinal cancer and precancerous lesions. Outcome information was obtained through standardized face-to-face interviews conducted by trained investigators and verified against previous endoscopic and pathological diagnoses from medical institutions. Diagnostic criteria were based on established national guidelines for upper gastrointestinal cancer and precancerous lesions. The composite outcome included esophageal or gastric cancer, esophageal or gastric intraepithelial neoplasia, Barrett's esophagus, cardia or gastric intestinal metaplasia, and severe chronic atrophic gastritis. These conditions were combined into a single outcome because they represent biologically related stages along the continuum of upper gastrointestinal carcinogenesis.

#### 2.2.2 Control group

According to the above standardized interviews, the control group consisted of study participants who self-reported no previous diagnosis of the above-mentioned upper gastrointestinal cancers or precancerous lesions by medical institutions and had no history of upper gastrointestinal malignancy.

### 2.3 Main exposure variable

The duration of household coal use for heating and cooking was the primary exposure variable in this study and was used to reflect the level of long-term cumulative household coal exposure. The data were obtained from the standardized structured questionnaire used in the upper gastrointestinal cancer early screening program of this study and were collected through face-to-face interviews conducted by uniformly trained investigators. To reduce recall bias and the risk of exposure misclassification, cumulative duration of use was categorized into three ordered groups: < 10 years (reference group), 10–19 years, and ≥20 years, and participants directly selected the category according to their actual household use situation.

### 2.4 Covariates

The study variables were derived from the upper gastrointestinal tumor screening risk assessment questionnaire. Based on previous literature and the study objectives, the following covariates were selected for analysis:

(1) Demographic variables: age (continuous variable, years), sex (male/female, binary variable), and region (Lucheng District/Pingshun County, binary variable);

(2) Variables related to previous disease history: first-degree family history of upper gastrointestinal tumors (yes/no, binary variable) and history of ulcer or perforation (yes/no, binary variable);

(3) Lifestyle-related variables: smoking history, alcohol drinking history, rapid eating, consumption of hot food, high-salt diet, and drinking water source. Among these, smoking history was defined as a binary variable and referred to continuous or cumulative smoking for ≥6 months during the lifetime, categorized as yes or no, including both current smokers at the time of the survey and former smokers with a previous smoking history, in order to avoid exposure misclassification bias. Alcohol drinking history and rapid eating were defined as binary variables (“yes/no”). Frequency-related variables, such as consumption of hot food and high-salt diet, were categorized into five levels: “never or rarely,” “1–3 days/month,” “1–3 days/week,” “4–6 days/week,” and “every day.” Drinking water source was categorized into four types: “1 tap water (treated), 2 deep well water/spring water, 3 lake water/river water, and 4 cellar water/pond water/shallow well water.”

The above covariates were mainly used to control for the influence of potential confounding factors, thereby improving the stability and reliability of the model estimates.

### 2.5 Sample size calculation

This study primarily used multivariable logistic regression models to analyze the association between household coal exposure and upper gastrointestinal cancer and precancerous lesions. To evaluate the adequacy of the sample size for the multivariable regression models, the events per variable (EPV) principle, which is widely recognized in the field of international epidemiology, was applied for validation (11, 12). According to this principle, each additional independent variable included in a regression model should correspond to at least 10 outcome events in order to ensure the stability of parameter estimation and to avoid the risk of overfitting. A total of 13 independent variables were ultimately included in the fully adjusted model of this study, corresponding to 783 outcome events. The resulting EPV was well above the recommended threshold of 10, suggesting adequate stability of the parameter estimates in the primary model.

### 2.6 Statistical analysis

Statistical analyses were performed using SPSS version 26.0 software. Normally distributed continuous variables were described as $\bar{\text{x}}$ ± s, and comparisons between groups were conducted using the independent-samples *t*-test. Non-normally distributed continuous variables were described as M (IQR), and comparisons between groups were conducted using the Mann–Whitney U-test. Categorical variables were described as *n* (%), and comparisons between groups were conducted using the χ^2^ test; Fisher's exact test was used when the expected frequencies were insufficient. Logistic regression models were used to analyze the association strength between household coal exposure and upper gastrointestinal cancer and precancerous lesions, and odds ratios (ORs) and their 95% confidence intervals (95% CIs) were calculated. Stratified subgroup analyses were conducted according to sex, age, region, smoking status, and alcohol consumption. Effect modification was assessed using interaction tests. Sensitivity analyses were performed to examine the robustness of the observed associations. All statistical tests were two-sided, and statistical significance was defined as a *P*-value < 0.05.

## 3 Results

### 3.1 Screening and inclusion of study participants

**Figure 1:**
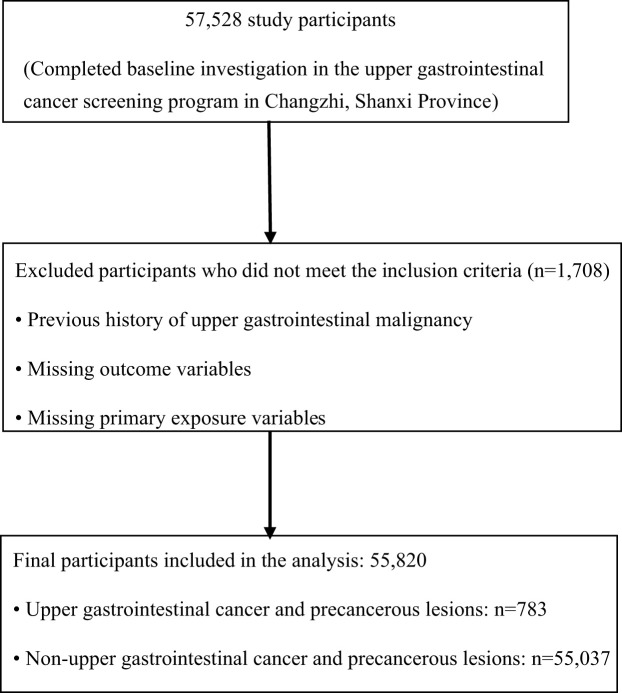
Flowchart of participant selection.

A total of 57,528 participants were surveyed. After excluding 1,708 participants who did not meet the inclusion criteria or had missing key information, 55,820 participants were ultimately included in the analysis. The flowchart of participant screening is shown in Figure 1.

### 3.2 Comparison of baseline characteristics between study participants

A total of 55,820 participants were included in the study, including 783 cases with upper gastrointestinal cancer and precancerous lesions and 55,037 controls. The mean age of the case group was higher than that of the control group [(64.00 ± 6.69) years vs. (60.46 ± 7.01) years, *P* < 0.05]. There were statistically significant differences between the case group and the control group in the distributions of sex and region (all *P* < 0.05). The proportions of participants with a first-degree family history of upper gastrointestinal tumors and a history of ulcer or perforation were significantly higher in the case group than in the control group (all *P* < 0.05). Statistically significant differences were also observed between the case group and the control group in multiple lifestyle factors and the distribution of household coal use duration (all *P* < 0.05). The detailed results are presented in Table 1.

**Table 1:** Comparison of baseline characteristics between study participants.

Variable	Case group	Control group	*t/χ^2^* value	*P*-value
Age	64.00 ± 6.69	60.46 ± 7.01	14.02	< 0.05
Sex			68.29	< 0.05
Male	506 (64.6)	27,382 (49.8)		
Female	277 (35.4)	27,655 (50.2)		
Region			205.00	< 0.05
Pingshun County	670 (85.6)	33,246 (60.4)		
Lucheng District	113 (14.4)	21,791 (39.6)		
Family_history			678.12	< 0.05
Yes	161 (20.6)	1,806 (3.3)		
No	622 (79.4)	53,231 (96.7)		
History of ulcer or perforation			1,907.74	< 0.05
Yes	78 (10.0)	136 (0.2)		
No	705 (90.0)	54,901 (99.8)		
Smoking history			52.76	< 0.05
Yes	276 (35.2)	13,237 (24.1)		
No	507 (64.8)	41,800 (75.9)		
Alcohol drinking history			23.36	< 0.05
Yes	173 (22.1)	8,665 (15.7)		
No	610 (77.9)	46,372 (84.3)		
Consumption of hot food			118.11	< 0.05
Don't eat or eat very little	606 (77.4)	46,231 (84.0)		
1–3 days per month	83 (10.6)	5,641 (10.2)		
1–3 days per week	22 (2.8)	1,622 (2.9)		
4–6 days per week	12 (1.5)	356 (0.6)		
Every day	60 (7.7)	1,187 (2.2)		
High-salt diet			193.93	< 0.05
Don't eat or eat very little	619 (79.1)	45,486 (82.6)		
1–3 days per month	92 (11.7)	6,511 (11.8)		
1–3 days per week	22 (2.8)	2,243 (4.1)		
4–6 days per week	12 (1.5)	426 (0.8)		
Every day	38 (4.9)	371 (0.7)		
Rapid eating			51.25	< 0.05
Yes	140 (17.9)	5,550 (10.1)		
No	643 (82.1)	49,487 (89.9)		
Drinking water source			-^a^	< 0.05
Tap water (treated)	619 (79.1)	46,658 (84.8)		
Deep well water, spring water	146 (18.6)	7,219 (13.1)		
Lake water, river water	0 (0.00)	47 (0.1)		
Underground cellar water, pond water, shallow well water	18 (2.3)	1,113 (2.0)		
Household coal use duration			50.57	< 0.05
< 10 years	256 (32.7)	24,740 (45.0)		
10–19 years	172 (22.0)	11,000 (20.0)		
≥20 years	355 (45.3)	19,297 (35.1)		

^a^Fisher's exact test was used for drinking water source because the expected frequencies in some cells were < 5.

### 3.3 Multivariable analysis of household coal use duration and upper gastrointestinal cancer and precancerous lesions

Multivariable logistic regression analysis showed that the duration of household coal use for cooking and heating was positively associated with upper gastrointestinal cancer and precancerous lesions. When modeled as an ordinal variable (< 10 years, 10–19 years, and ≥20 years), each increment in exposure category was associated with a 13% increase in the odds of upper gastrointestinal cancer and precancerous lesions (OR = 1.13, 95% CI: 1.05–1.24; *i* for trend = 0.003). Age, first-degree family history of upper gastrointestinal cancer, history of ulcer or perforation, consumption of hot foods, high-salt diet, and duration of household coal use were significantly associated with the outcome (all *P* < 0.05). Sex and region were inversely associated with the outcome (both *P* < 0.05). The detailed results are presented in Table 2.

**Table 2:** Multivariable logistic regression analysis of household coal use duration and upper gastrointestinal cancer and precancerous lesions.

Variables	β	SE	OR	95% CI	*P*-value
Age	0.07	0.005	1.07	1.06–1.08	< 0.001
Sex	−0.54	0.078	0.58	0.50–0.67	< 0.001
Region	−1.16	0.107	0.31	0.25–0.39	< 0.001
Family_history	1.67	0.099	5.32	4.38–6.45	< 0.001
History of ulcer or perforation	3.59	0.163	36.16	26.28–49.74	< 0.001
Consumption of hot food	0.18	0.037	1.20	1.12–1.29	< 0.001
High-salt diet	0.14	0.046	1.15	1.05–1.25	0.003
The duration of household coal use	0.13	0.043	1.13	1.05–1.24	0.003

All binary variables were coded as “exposure group = 1” and “control group = 0.” The reference groups were male sex, Pingshun County, no family history, no history of ulcer or perforation, and never or rarely consuming hot food/high-salt diet, respectively. The duration of household coal use for heating and cooking was treated as an ordinal categorical variable and coded as < 10 years = 1, 10–19 years = 2, and ≥20 years = 3, with the < 10 years group as the reference group. Age was treated as a continuous variable, with years as the unit, and the OR value represented the change in risk associated with each 1-year increase in age.

### 3.4 Subgroup analysis

To assess potential heterogeneity in the association between household coal use duration and upper gastrointestinal cancer and precancerous lesions, stratified analyses were performed according to sex, age, region, smoking status, and alcohol consumption. All subgroup models were adjusted for the same covariates as the primary model, and interaction tests were conducted. Positive associations were observed across all subgroups, with statistically significant associations identified among women, participants aged ≥65 years, non-smokers, non-drinkers, and residents of Pingshun County (all *P* < 0.05). The remaining subgroup estimates were in a similar direction but did not reach statistical significance, which may be attributable to limited statistical power. Importantly, no significant interactions were observed for any subgroup variable (all *P* for interaction > 0.05), indicating no evidence that the association differed across subgroups. Therefore, these subgroup findings should be interpreted as exploratory and hypothesis-generating. Detailed results are shown in Table 3.

**Table 3:** Subgroup analysis of the association between duration of household coal use and upper gastrointestinal cancer and precancerous lesions.

Subgroup variable	Subgroup category	OR (95% CI)	*P*-value	Interaction *P*-value
Sex	Male	1.122 (1.010–1.247)	0.032	0.664
	Female	1.187 (1.029–1.368)	0.018	
Age	< 65 years	1.130 (1.003–1.274)	0.044	0.301
	≥65 years	1.168 (1.035–1.317)	0.011	
Smoking	With smoking history	1.134 (0.981–1.310)	0.089	0.654
	Without smoking history	1.160 (1.045–1.287)	0.005	
Alcohol drinking	With alcohol drinking history	1.126 (0.940–1.349)	0.199	0.697
	Without alcohol drinking history	1.154 (1.049–1.270)	0.003	
Region	Pingshun County	1.152 (1.051–1.263)	0.002	0.928
	Lucheng District	1.133 (0.906–1.416)	0.273	

### 3.5 Sensitivity analysis

To verify the robustness of the association between the duration of household coal use for heating and cooking and upper gastrointestinal cancer and precancerous lesions, this study conducted analyses using progressive multivariable logistic regression models with the stepwise inclusion of confounding factors from different dimensions. The results are shown in Table 4. A total of five progressive models were constructed. Starting from a crude model including only the core exposure, demographic characteristics, disease history, lifestyle factors, dietary habits, and other potential confounding factors were sequentially included, ultimately forming a fully adjusted model. Linear trend tests were simultaneously performed for all models. The results showed that, across all models, the positive association between the duration of household coal use for heating and cooking and disease risk remained completely consistent in direction. The risks in the 10–19 years group and the ≥20 years group both remained statistically significant, and the dose–response relationship was stable. In the fully adjusted model, the disease risk increased by 38.3% in the 10–19 years group (OR = 1.383, 95% CI: 1.129–1.694, *P* = 0.002) and by 33.0% in the ≥20 years group (OR = 1.330, 95% CI: 1.120–1.580, *P* = 0.001), with a linear trend test result of *P* = 0.001. These findings indicate that the positive association between household coal use duration and upper gastrointestinal cancer and precancerous lesions remained consistent across models with different levels of adjustment, supporting the robustness of the study findings.

**Table 4:** Sensitivity analysis of household coal use duration and upper gastrointestinal cancer and precancerous lesions.

Model type	< 10 years	10–19 years		≥**20 years**		Trend test *P*-value
	OR (95% CI)	OR (95% CI)	*P*-value	OR (95% CI)	*P*-value	
Model 1 Crude model	1.00	1.511 (1.244–1.836)	< 0.001	1.778 (1.512–2.090)	< 0.001	< 0.001
Model 2 Demographic-adjusted model	1.00	1.355 (1.114–1.649)	0.002	1.373 (1.165–1.618)	< 0.001	< 0.001
Model 3 Disease history-adjusted model	1.00	1.371 (1.120–1.677)	0.002	1.288 (1.086–1.527)	0.004	0.005
Model 4 Lifestyle-adjusted model	1.00	1.374 (1.123–1.682)	0.002	1.289 (1.086–1.529)	0.004	0.005
Model 5 Fully adjusted model	1.00	1.383 (1.129–1.694)	0.002	1.330 (1.120–1.580)	0.001	0.001

Model 1 included only the core exposure variable, namely the duration of household coal use for heating and cooking. Model 2 was adjusted for age, sex, and region based on Model 1. Model 3 was additionally adjusted for first-degree family history of upper gastrointestinal tumors and history of ulcer or perforation based on Model 2. Model 4 was additionally adjusted for smoking status and alcohol drinking status based on Model 3. Model 5 was additionally adjusted for consumption of hot food, high-salt diet, rapid eating, drinking water source, and pickled food based on Model.

## 4 Discussion

In this cross-sectional study, we observed a positive association between the duration of household coal use and upper gastrointestinal cancer and precancerous lesions after adjustment for age, sex, region, family history, and relevant lifestyle factors. This association remained generally consistent across models with different levels of covariate adjustment and in sensitivity analyses, supporting the robustness of the findings. These results suggest that long-term household coal use may be associated with upper gastrointestinal cancer and precancerous lesions and warrant further investigation in high-incidence areas such as Shanxi Province.

This study adopted a composite outcome design combining upper gastrointestinal cancer and precancerous lesions, which has sufficient scientific basis and public health value. First, all included lesions are recognized by authoritative domestic guidelines as precancerous lesions/states of upper gastrointestinal cancer and have clear biological continuity with the carcinogenic process, thereby covering the entire disease progression spectrum. Second, this design is consistent with the core prevention and control strategy of early screening, early diagnosis, and early treatment for upper gastrointestinal cancer in China and is suitable for primary-level prevention and control needs in high-incidence areas. Third, it increases the number of outcome events, thereby ensuring statistical power and avoiding type II errors caused by the low incidence of cancer in the general population.

The findings of this study are generally consistent with those of multiple previous domestic and international studies. Josyula et al. (13) found that long-term exposure to indoor air pollution related to solid fuel combustion was associated with an increased risk of non-pulmonary cancers, including upper gastrointestinal cancer. Sapkota et al. (10) also reported that the use of coal for cooking may increase the levels of multiple potential carcinogens in indoor air, thereby increasing long-term health risks in the population. Patel et al. (14), in exploring the multifactorial etiology of esophageal cancer, also mentioned that household energy structure and coal-related environmental exposure may be involved in its pathogenesis. In Chinese populations, a prospective cohort study based on approximately 500,000 Chinese adults showed that long-term exposure to solid cooking fuel combustion was associated with an increased risk of esophageal cancer (15). Tian et al. (16) further reported that the use of coal or other solid fuels for cooking was associated with all-cause mortality and cancer mortality, suggesting that household coal-related exposure may affect multiple cancer outcomes in the Chinese population. Compared with the above studies, the present study further refined household coal exposure from simple use/non-use status to duration of use and simultaneously focused on upper gastrointestinal cancer and precancerous lesions in the general population. The findings suggest that a positive association may exist between the duration of household coal use and related diseases. On the basis of confirming the above findings, this study provides supplementary epidemiological evidence for further exploring the association between the two.

Household coal exposure is not the effect of a single pollutant but rather a complex exposure process involving multiple pollutants, mainly including carbon monoxide (CO), nitrogen dioxide (NO_2_), particulate matter (PM), and polycyclic aromatic hydrocarbons (PAHs). In some economically underdeveloped areas of China, households using solid fuels for cooking or heating often lack chimneys or have limited ventilation conditions, resulting in long-term exposure of residents to relatively high levels of coal smoke pollution (6). Previous studies have shown that chemical substances produced by coal combustion, such as PAHs, can accumulate in indoor environments and enter the human body through the respiratory and digestive tracts, thereby increasing both external and internal exposure burdens to harmful pollutants (17). At the biological mechanism level, Jeong et al. (18) reported that PAHs, as important components of particulate matter, may participate in the occurrence of gastrointestinal diseases by inducing inflammatory responses and damaging the gastrointestinal mucosa and may even exhibit potential carcinogenic effects. Meanwhile, Nagel et al. (19), based on analyses of multiple European cohorts, further indicated that long-term exposure to PM2.5 and NO_2_ may participate in the occurrence and progression of upper gastrointestinal tumors through multiple mechanisms, including oxidative stress and inflammatory responses, genetic and epigenetic alterations, and changes in intracellular homeostasis and microbiota interactions. In studies conducted in Chinese populations, evidence has also shown that long-term exposure to air pollutants such as particulate matter, SO_2_, and nitrogen oxides is significantly associated with digestive system diseases (20). In addition, a large prospective cohort study from the United Kingdom found that long-term (5-year) exposure to higher concentrations of NO_2_ was associated with an increased risk of esophageal cancer (21).

This study has several limitations. First, owing to its cross-sectional design, the temporal relationship between household coal use duration and upper gastrointestinal cancer and precancerous lesions could not be established. Therefore, the findings should be interpreted as associations rather than evidence of causality. In addition, reverse causation cannot be excluded, as household energy-use patterns may have changed following disease diagnosis. Second, information on household coal use was obtained through self-report and categorized solely according to duration of use. Detailed exposure characteristics, including stove type, ventilation conditions, fuel-use intensity, and mixed fuel use, were not available. Consequently, the exposure measure may not accurately reflect individual exposure levels. Given the relatively older age of the study population, recall-based reporting of coal use duration may also have introduced exposure misclassification and recall bias. Third, although multiple potential confounders were adjusted for in the analyses, several important factors, including socioeconomic status, occupational exposures, passive smoking, body mass index, Helicobacter pylori infection, and fruit and vegetable intake, were not available because of questionnaire limitations. Therefore, residual confounding cannot be completely excluded. Fourth, Subgroup analyses showed statistically significant associations in certain strata, including those defined by sex, age, smoking status, alcohol consumption status, and residence in Pingshun County. However, none of the interaction tests reached statistical significance, indicating insufficient evidence for meaningful differences in effect estimates across subgroups. Therefore, these findings should be considered exploratory and hypothesis-generating and warrant further investigation in larger populations. Fifth, a composite outcome comprising upper gastrointestinal cancer and precancerous lesions was used. Although this approach increased the number of outcome events and improved statistical power, the strength of association between household coal use and individual disease entities may differ. We were unable to perform separate analyses for cancer and precancerous lesions or evaluate potential heterogeneity across specific disease types. Furthermore, outcome ascertainment was based on previous endoscopic and pathological diagnoses, which may have been influenced by healthcare utilization, health awareness, and screening behaviors. Because information on prior screening history and socioeconomic factors was unavailable, the possibility of detection bias and residual confounding cannot be completely ruled out.

In conclusion, this study identified a positive association between the duration of household coal use and upper gastrointestinal cancer and precancerous lesions. Given that Shanxi Province is both a high-incidence area for upper gastrointestinal cancer and a region where household coal use remains prevalent, these findings may be of public health interest. However, due to the cross-sectional nature of the study, causal inferences cannot be established. Future prospective studies with more detailed exposure assessment are needed to confirm these findings and further clarify the potential role of household coal exposure in the development of upper gastrointestinal cancer and precancerous lesions.

## Data Availability

The raw data supporting the conclusions of this article will be made available by the authors, without undue reservation.
